# Efficacy of Liraglutide in clinical practice: Single centre experience

**DOI:** 10.12669/pjms.36.3.358

**Published:** 2020

**Authors:** Muhammad Owais Rashid, Sumerah Jabeen, Adeel Khoja, Najmul Islam

**Affiliations:** 1 M. Owais Rashid MBBS, FCPS (Medicine), FCPS (Endocrinology), Consultant Endocrinologist, Section of Endocrinology, Department of Medicine, Aga Khan University Hospital, Karachi, Pakistan; 2 Sumerah Jabeen, FCPS (Medicine). Fellowship in Diabetes & Endocrinology, Aga Khan University Hospital, Karachi, Pakistan; 3 Adeel Khoja, MBBS. Senior Instructor Research, Department of Medicine, Aga Khan University Hospital, Karachi, Pakistan; 4 Najmul Islam, MBBS, FRCP. Consultant Endocrinologist, Section Head Endocrinology, Department of Medicine, Aga Khan University Hospital, Karachi, Pakistan

**Keywords:** Glucagon like peptide-1(GLP-1) agonist, Liraglutide, Type 2 diabetes

## Abstract

**Background and Objective:**

GLP-one receptor agonists are amongst the unique antidiabetes medications that have significant metabolic and cardiovascular benefits in addition to glucose lowering effect. To best of our knowledge, there is no published data on efficacy of liraglutide use among Pakistani population.Our objective was to ascertain the efficacy of liraglutide use among type two diabetes patients.

**Methods:**

This retrospective study was conducted at the Endocrinology Clinics of Aga Khan University Hospital (AKUH) Karachi, Pakistan during the period from July 01, 2016 to 30th June, 2017. Liraglutide was prescribed to 68 obese type two diabetes patients with uncontrolled diabetes taking more than one oral medication ± insulin. Starting dose of Liraglutide was 0.6 mg, which was increased to 1.2 mg after 1-2 weeks with further increment to 1.8 mg/day based on tolerance and individual patient preference. Dose of other diabetes medications was adjusted according to clinical judgment whereas Dipeptidyl peptidase-4(DPP-4) inhibitors were discontinued.

**Results:**

Mean age of cohort was 55 years (SD=10.94 years) with median body mass index of 36.45 kg/m^2^ and majority (57.35%) were on a dose of 1.2 mg of Liraglutide per day. Median HbA_1_c reduced to 7.50% and 7.40% at three months and six months respectively vs 8.45% at baseline. Mean reduction in weight after three month was two kilograms and at six months, it was 1.38 kilograms respectively.

**Conclusion:**

Liraglutide as add on therapy demonstrated favourable HbA1c and weight reduction in obese uncontrolled type two Diabetes Pakistani subjects.

## INTRODUCTION

Type 2 diabetes being a complex metabolic disorder remains an ever-increasing global health care issue. While glycemic control is associated with reductions in the risk of micro vascular complications, the macro vascular benefits of glycemic control are not very clear.^1^ The reason might be that the benefits of improved glycemic control take a long time to influence the progression of atherosclerotic vascular disease.

There are multiple treatment options available for patients with diabetes, many of which are either weight neutral, cause weight gain or promote weight loss. One of the disadvantage of having these multiple treatment options is that, the wide range of options may cause problem for the health care provider to prescribe the optimal regimen. In making these decisions, the prescriber should consider patient’s disease progression, comorbidities, and concomitant treatment.^2^

After decades of disappointment in attempting to control cardiovascular disease progression in type 2 diabetes with careful glycemic control,a new found hope that newer drugs, particularly the Glucagon like peptide 1 receptor (GLP-1) agonist and the sodium glucose co transporter 2 SGLT-2 inhibitors have cardiovascular benefits independent of glycemic control has led to a new dimension in the management of type 2 diabetes.^3^ Despite its role in glucose homeostasis, the GLP-1 receptor is amazingly widely distributed throughout the body, including heart. GLP-1 may exert its effects through both receptor-dependent and receptor-independent mechanism and through the actions of both the intact peptide and its metabolites.^4^ Injectable GLP-1 receptor agonist mimics endogenous GLP-1 by stimulating pancreatic insulin secretion. They have beneficial nonglycemic metabolic effects, notably the potential for weight loss.^5^

Liraglutide a long acting GLP-1 receptor agonist has been approved for the treatment of type 2 diabetes.^6^ To assess the long-term effects of liraglutide on cardiovascular outcomes and other clinically important events, the Liraglutide Effect and Action in Diabetes: Evaluation of Cardiovascular Outcome Results (LEADER) trial was initiated in 2010.^1^ In the LEADER’s trial, 9340 patients with type 2 diabetes at high cardiovascular risk were randomized to receive the longer-acting drug liraglutide 1.8 mg (or maximum tolerated dose) or matching placebo once daily and followed for a mean of 3.5 years. There was a 23% statistically significant (*p*-value= 0.001 for non-inferiority, *p*-value=0.01 for superiority) reduction in the primary end point, including a 22% reduction in cardiovascular mortality (*p*-value=0.007) and 15% reduction in total mortality (*p*-value=0.02).^3^ Gastrointestinal adverse effects were more common in the patients randomized to liraglutide, the trial concluded that the patients in liraglutide group had significantly reduced risk of cardiovascular events and they had better micro vascular outcomes then the group with standard therapy.^1^ A few studies have been conducted on South Asian population in India and all of these studies concluded that, liraglutide significantly improves glycemic control with low risk of hypoglycemia and is associated with significant weight loss in Indian patients with type 2 diabetes mellitus.^7^ To best of our knowledge, there is no published data on efficacy of liraglutide use among Pakistani population.

Hence, this study aimed to assess the efficacy of liraglutide in terms of glycemic control and weight reduction in type 2 diabetes patients Pakistani population during routine clinical practice at outpatient clinic.

## METHODS

This retrospective study was conducted at the Endocrinology Clinics of Aga Khan University Hospital (AKUH) Karachi, Pakistan during the period from July 01, 2016 to 30th June, 2017. This study was approved by the ethical committee (Ref. No. 4911-Med-ERC-17) of the Aga Khan University.

Liraglutide was prescribed to 68 obese (BMI >23Kg/m^2^) type 2 diabetes patients with uncontrolled diabetes (HbA1C>7%). All patients were on more than one oral hypoglycemic agents with or without insulin. DPP4 inhibitors were stopped if patients were on it whereas other antidiabetes medication were adjusted according to clinical judgment. All patients were advised to start 0.6 mg/day of liraglutide. An increment in dose up to 1.2 mg/day was done after 1-2 weeks which was later increased to 1.8 mg/day based on tolerance and individual patient preference.

Patients aged below 16 years, non diabetics, having type 1 diabetes, not taking liraglutide were excluded from the study. Informed written consent was obtained from hospital authorities to use the data of patients. No personal detail by any means related to any patient was revealed in regard of ethical considerations.

Data of patients taking liraglutide was retrieved from hospital medical record maintained by Health Information Management System of Aga Khan University Hospital after acquiring permission. Data collection was done by means of a data collection tool specifically designed for the study, keeping in mind the indicators/variables asked from the diabetes patient at the time of initial and follow-up visits in the diabetes clinic. A research assistant was trained to review the files of all the diabetes patients falling under the aforementioned timelines by means of a data collection tool. Data collection tool mainly covered information on demographic details of the patient, duration of diabetes and patients with history of Hypertension (taking anti hypertensive medication or BP>140/90mmhg on home BP monitoring), Dyslipidemia (serum LDL>100 mg/dl, triglycerides >150mg/dl), Coronary artery disease including history of unstable angina, previous history of Myocardial infarction or coronary angioplasty, retinopathy (both proliferative and non proliferative) diagnosed and treated by ophthalmologist, neuropathy (diagnosed on previous visits with compromised vibration and pin prick sensation assessed with a calibrated tuning fork and 10gm monofilament testing) and nephropathy (confirmed by presence of albuminuria in urine on 2 or more occasions), medication details, changes in glycosylated haemoglobin(HbA1C) and weight, any reported side effects of liraglutide and history of discontinuation if any.

Graphical Representation of all Continuous Variables in the Dataset

**Figure:**
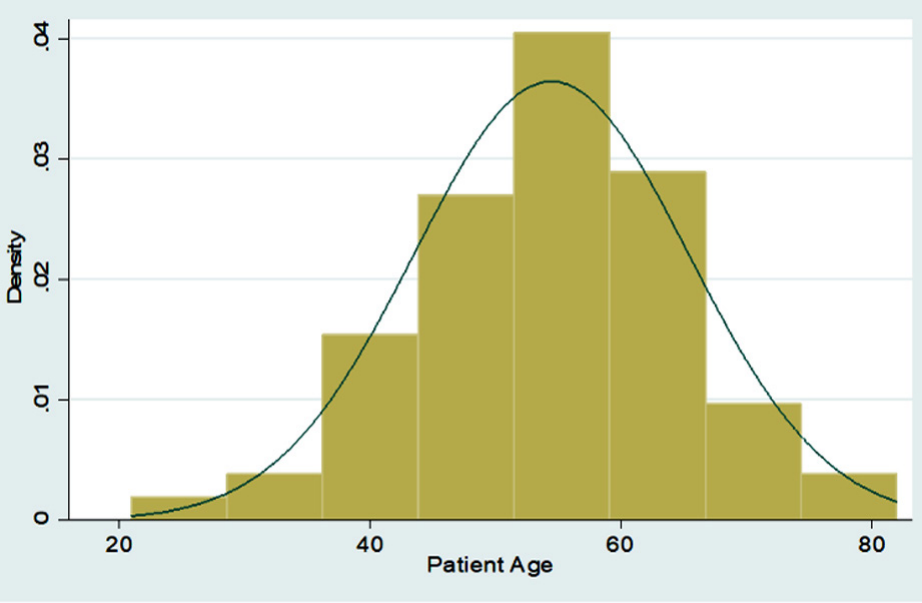
Histograms: AGE Distribution Check: Symmetrically Distributed.

**Figure:**
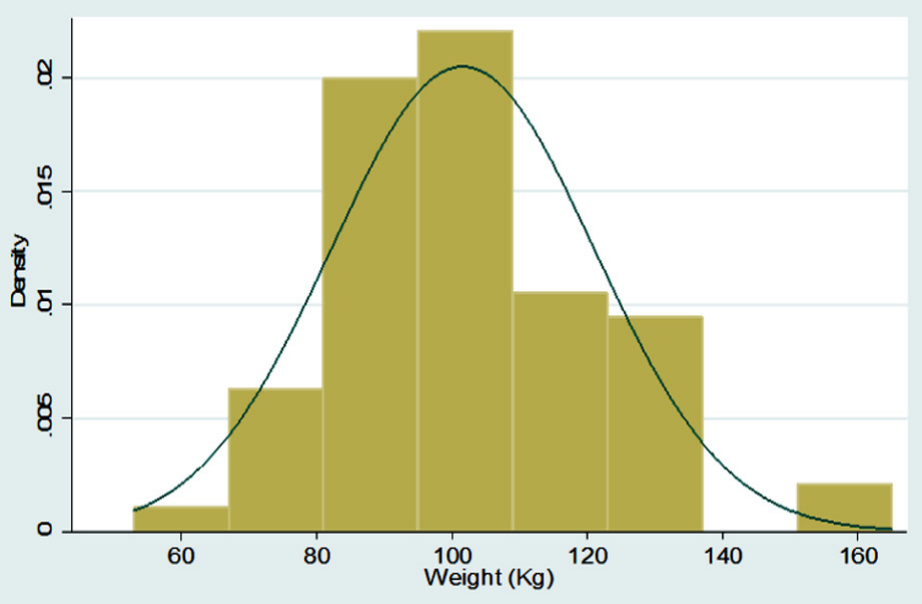
WEIGHT: Distribution Check: Symmetrically Distributed.

**Figure:**
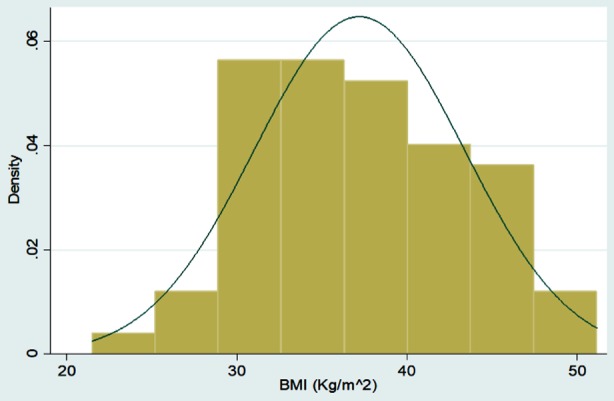
BODY MASS INDEX (BMI): Distribution Check: Asymmetrically Distributed.

**Figure:**
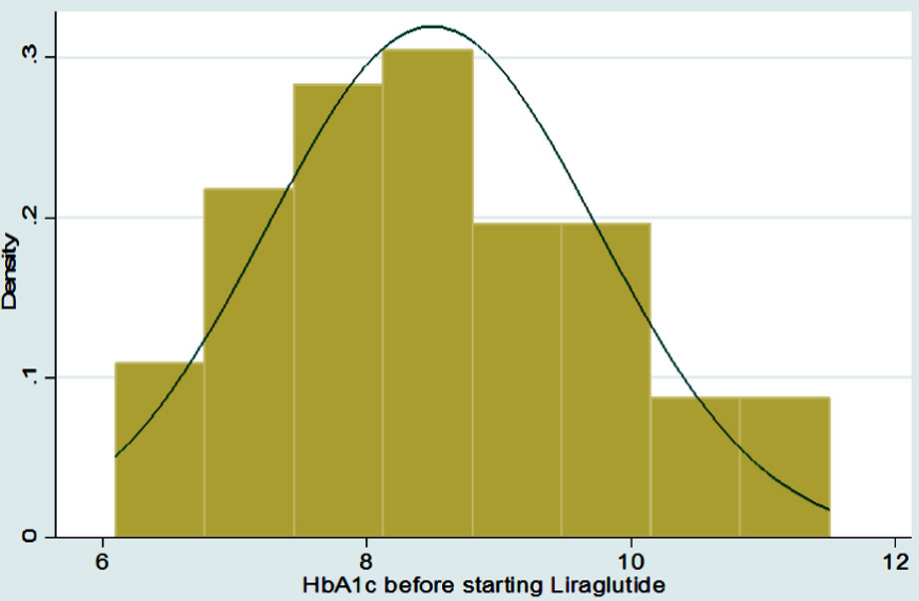
HBA1C BEFORE STARTING LIRAGLUTIDE: Distribution Check: Asymmetrical Distribution.

### Plan of Analysis

Frequencies with percentages were reported for categorical variables for e.g. gender, associated comorbids, and associated Diabetes complications, medication history before adding liraglutide, side effects of Liraglutide etc. Mean with standard deviation was reported for all symmetrically distributed continuous variables while median with inter quartile range was reported for all asymmetrically distributed variables for e.g. age, duration of comorbids, baseline anthropometric measurements such as height, weight, BMI, BP, Pulse, HbA1C and subsequent followup measurements at three and six months, etc. All the analysis was performed on STATA version 12.

## RESULTS

Of the total 68 patients studied, mean age of cohort was 55 years (SD=10.94 years). Among them 50% were male, having a median diabetes duration of 13 years. More than four-fifth of our study population had dyslipidemia and hypertension and less than 15% had coronary artery disease (Table-I). Median body mass index of our study participants was 36.45 kg/m^2^, and majority of them (57.35%) were on a dose of 1.2 mg of Liraglutide per day. Study participants were mostly on maximum doses of sulfonylureas (44%), metformin (94%), DPP-4 inhibitors (49%), basal insulin (31%), pre-mixed insulin (28%), basal bolus regimen (32%) either alone or in combination before addition of Liraglutide to the treatment regimen. DPP-4 inhibitors were discontinued in all patients once liraglutide was introduced and dosage of other antidiabetes medications adjusted. Median HbA_1_c at baseline was 8.45% and it reduced to 7.50% and 7.40% at three months and six months post-Liraglutide respectively. The mean reduction in weight after three months of Liraglutide was 2 kilograms and at six months, it was 1.38 kilograms respectively. There was no significant difference noted in the systolic and diastolic blood pressures and the pulse rate at three and six months from the baseline, respectively (Table-I).

**Table-I:** Descriptive analysis of patients on liraglutide.

S.No	Variables	n = 68 (%)
1	Age*	54.47 ± 10.94
2	Gender	
	(a)Male	b34 (50%)
	(b)Female	34 (50%)
3	Duration of Diabetes*	13 ( 7 – 19)
4	Presence of Dyslipidemia	57 (83.82%)
5	Presence of Hypertension	61 (89.71%)
6	Presence of Coronary Artery Disease	9 (13.24%)
7	Presence of Retinopathy	4 (5.90%)
8	Presence of Neuropathy	5 (7.35%)
9	Presence of Nephropathy	3 (4.41%)
10	Body Mass Index (kg/m^2^)*	36.45 (32.40 – 41.9)
11	Dose of Liraglutide (mg)	
	(a)0.6	77 (10.29%)
	(b)1.2	39 (57.35%)
	(c)1.8	22 (32.35%)
12	Participants on Sulfonylurea before liraglutide	30 (44.12%)
13	Participants on Metformin before liraglutide	64 (94.12%)
14	Participants on Thiazolidinedione before liraglutide	4 (5.88%)
15	Participants on DDP-4 inhibitors before liraglutide	33 (48.53%)
16	Participants on Basal Insulin before liraglutide	22 (30.88%)
17	Participants on Pre-Mixed Insulin before liraglutide	19 (27.94%)
18	Participants on Basal Bolus Regimen before liraglutide	22 (32.35%)
19	Creatinine levels of participants *	0.8 (0.7 – 0.95)

**Table-II:** Comparison of variables from baseline following Liraglutide treatment.

S.No	Variables	At Baseline	At 3 months	At 6 months
1	HbA1c (at baseline) of participant (%)*	8.45 (7.55 – 9.45)	7.50 (6.70 – 8.6)	7.40 (6.70 – 7.90)
2	Change in weight after three months post-Liraglutide of participants*	-	-2.00±3.10	-1.38 ± 3.27
3	Systolic Blood Pressure (at baseline) of participants (mmHg)*	138.28 ± 17.11	134.95 ± 23.60	138.07 ± 15.60
4	Diastolic Blood Pressure (at baseline) of participants (mmHg)*	74.42 ± 10.05	72.93 ± 8.80	72.64 ± 11.70
5	Pulse Rate (at baseline) of participants (beats/min)*	89.34 ± 13.50	90.40 ± 14.85	92.14 ± 16.61
6	Side Effects reported by participants post-Liraglutide^$^:			
	1.Nausea		6 (8.82%)	
	2.Vomiting		4 (5.88%)	
	3.Diarrhea		5 (7.35%)	
	4.Others		8 (11.76%)	
7	Liraglutide discontinued due to side effects & cost		7 (10.29%)	

Side-effects reported after the start of Liraglutide were mainly nausea (9%), vomiting (6%), diarrhoea (7%) and others (12%) which included headache, dizziness and constipation. In addition to this, Liraglutide was discontinued due to the side effects and cost in about seven participants (10%) of our study cohort and therefore they were not included in the final analysis.

## DISCUSSION

This small scale study verifies that Liraglutide as an add on therapy was beneficial for both HbA1c and weight reduction in Pakistani subjects with Diabetes Mellitus (T2DM). The HbA1c reduction at 3 months from baseline in our study was 0.95% and 1.05% at 6 months. There was -2.00 ± 3.10 kg weight reduction at three months and -1.38 ± 3.27 kg at 6 months. The HbA1c reduction in our study corresponds to the maximum HbA1c reduction noted in LEAD -4 trial which showed 1.5 % reduction at 1.2 and 1.8 mg of Liraglutide doses at 24 weeks of treatment.^8^ Although the mean weight reduction in our study noted at six months of treatment was less as anticipated but it was comparable to weight reduction in LEAD trials which was also less than 3 kg with the exception of LEAD-6 which showed a weight reduction of 3.24 kg.^9^ The probable explanation to this could be that weight loss demonstrated with Liraglutide is dose dependant whereas in our study more than 50% subjects were on 1.2mg doses.^10^-^12^ No change in systolic, diastolic blood pressures and pulse at baselines and at six months post treatment from the baseline was noted although this was not the primary objective and includes several confounding factors like retrospective design, medication adherence, operator variability etc. Gastrointestinal side effects were the commonly reported adverse effects among the study participants and seven participants withdrew liraglutide owing to these side effects. Financial constraints remained an important factor in continuing patients on Liraglutide therapy.

Following the LEADER trial which showed significant reduction in the composite outcome of the first occurrence of death from CV causes, non-fatal myocardial infarction or nonfatal stroke in T2DM subjects with high CV risk, Liraglutide along with other GLP 1 agonists is now considered as second line therapy in high risk CV patients. The EASD and the ADA updated 2018 consensus statement for DM management in patients with T2 DM encourages use of GLP 1 receptor agonist and SGLT2 inhibitor for patients with clinical cardiovascular disease as an add on therapy after metformin.^13^ The exact mechanism influencing the beneficial effects of Liraglutide on cardiovascular benefit and weight reduction remains unknown but one probable hypotheses is reduction in visceral fat, hepatic steatosis and systemic inflammation which are considered strong contributors for cardiovascular morbidity in patients with diabetes.^14^,^15^ Bouchi et al. demonstrated the Liraglutide efficacy in reducing visceral fat and thus ameliorating hepatic steatosis, albuminuria and microinflammation in T2DM patients along with improved quality of life following treatment.^16^

This single centre retrospective study is the first Pakistani study in evaluating the liraglutide efficacy in T2 Diabetes Pakistani subjects. The mean duration of diabetes in our study population was 13 years but nevertheless there was a mean reduction in HbA1c at both three months and six months. Kaur et al. in their Liraglutide trial highlighted that weight reduction and diabetes control was irrespective of the duration of diabetes and baseline HbA1c thus concluding that liraglutide effectiveness could be achieved in patients with long standing diabetes as well.^17^ Although favourable HbA1C and weight reduction was found at six months post liraglutide several confounding factors like diet and physical activity could not be controlled due to retrospective design of study.

Despite the effective HbA1c and weight reduction Liraglutide remains an expensive treatment option for a country like Pakistan where majority of the patients do not have medical insurance. Since this is a single centre experience, prospective studies are required evaluating multiple beneficial effects of liraglutide in patients with CV risks and baseline albuminuria thus evaluating the new cardio protective and renal protective role of this drug.

## CONCLUSION

Liraglutide as add on therapy demonstrated favourable HbA1c and weight reduction in a real life clinical setting of Pakistani Type-2 diabetes patients. Despite the efficacy, financial constraints remain an important set back in utilizing the maximum benefitting effects of this drug.

### Authors’ Contribution:

**MO** conceived the study.

**AK** helped in data analysis.

**MO and SJ** were involved in drafting of manuscript and is responsible for integrity of research.

**NI** did review of manuscript. All authors read and approved the final manuscript.
